# Study on the Influence and Mechanism of Steel, Polyvinyl Alcohol, and Polyethylene Fibers on Slag–Yellow River Sediment Geopolymers

**DOI:** 10.3390/polym17081072

**Published:** 2025-04-16

**Authors:** Ge Zhang, Enhui Jiang, Kunpeng Li, Huawei Shi, Chen Chen, Chengfang Yuan

**Affiliations:** 1 Yellow River Institute of Hydraulic Research, Yellow River Water Conservancy Commission, Zhengzhou 450003, China; gezhangyrihr@163.com (G.Z.); jiangenhui@hky.yrcc.gov.cn (E.J.); 15538352232@163.com (H.S.); 15617633649@163.com (C.C.); 2 Key Laboratory of Lower Yellow River Channel and Estuary Regulation, Ministry of Water Resources, Zhengzhou 450003, China; 3 Yellow River Laboratory, Zhengzhou 450003, China; 4 College of Civil Engineering, Zhengzhou University, Zhengzhou 450001, China

**Keywords:** Yellow River sediment (YRS), geopolymer, fiber, strength, microstructure

## Abstract

Steel fibers (STs), polyvinyl alcohol fibers (PVAs), and polyethylene fibers (PEs) were selected to systematically investigate the effects of different fiber types and dosages on the workability (slump and spread) and mechanical properties (compressive strength and splitting tensile strength) of slag–Yellow River sand geopolymer eco-cementitious materials. By combining microstructural testing techniques such as thermogravimetric-differential thermal analysis (TG-DTA), X-ray diffraction (XRD), and scanning electron microscopy-energy dispersive spectroscopy (SEM-EDS), the influence mechanisms of fibers on the characteristic products and microstructure of the matrix were thoroughly revealed, and the role of fibers in the strength development of Yellow River sediment-based geopolymers was elucidated. The results show that as the fiber content increases, the workability of the mixture significantly decreases. The appropriate incorporation of steel fibers and PVAs can significantly enhance the strength and toughness of the matrix. When the fiber dosage is 1%, the 28-day compressive strength of specimens with steel fibers and PVAs increased by 25.93% and 21.96%, respectively, compared to the control group, while the splitting tensile strength increased by 50.00% and 60.34%, respectively. However, the mechanisms of action differ significantly; steel fibers primarily enhance the compressive performance of the matrix through their high stiffness and strength, whereas PVAs inhibit crack propagation through their flexibility and excellent bonding properties. In contrast, the strength improvement of PEs is mainly reflected in toughening. When the fiber dosage is 1.5%, the 28-day splitting tensile strength of PE specimens increased by 72.61%, and the tensile-to-compressive ratio increased by 92.32% compared to the control group. Microstructural analysis indicates that the incorporation of different types of fibers does not alter the types of characteristic products in alkali-activated cementitious materials, but excessive fiber content affects the generation of gel-like products and the distribution of free water, thereby altering the thermal decomposition behavior of characteristic gel products. Additionally, the matrix incorporating PEs forms a honeycomb-like amorphous gel, resulting in weak interfacial bonding between the fibers and the matrix. This is one of the main reasons for the limited reinforcing effect of PEs at the microscopic scale and a key factor for their inferior long-term performance compared to steel fibers and PVAs. This study provides theoretical foundations and practical guidance for optimizing the performance of fiber-reinforced geopolymer materials.

## 1. Introduction

The protection of the Yellow River is crucial for the long-term revitalization of China. The Yellow River basin faces significant sedimentation challenges due to factors such as low water volume, excessive sediment, and imbalanced water–sediment relationships. Sediment deposition presents a critical technical challenge for flood control, maintaining reservoir storage capacity, and ensuring the safe operation of irrigation systems in the region. However, the demand for construction sand in China is enormous, with approximately 20 billion tons of sand and gravel used annually [1]. Consequently, natural sand resources are increasingly depleted, necessitating the search for stable alternatives. Against this backdrop, the resource attributes and economic value of Yellow River sediment have become prominent. The main component of Yellow River sediment is silicon dioxide (SiO_2_) and based on this component alone Yellow River sediment is a potential alternative material. Utilizing Yellow River sediment resources offers significant socio-economic and ecological environmental benefits.

Ordinary Portland cement (OPC) is the most commonly used cementitious material in the construction industry. Its extensive use has contributed to global infrastructure and economic development to a certain extent. However, the OPC production process causes significant harm to the natural environment [2]. This highlights the urgent need to develop low-carbon, high-performance cementitious materials for infrastructure needs. Geopolymers, also known as alkali-activated cementitious materials, are characterized by a rapid setting speed [3,4], high-temperature resistance [5,6,7], corrosion resistance [8,9,10], and acid resistance [11,12]. Geopolymers can be prepared without the need for calcination, offering great potential to reduce carbon emissions by replacing cement [13,14,15,16]. However, similar to conventional concrete, geopolymer materials suffer from high brittleness [17,18] and low tensile strength [19,20], which limits their widespread application [21,22].

Incorporating fibers into geopolymer materials can significantly enhance their engineering properties, such as their tensile strength, flexural strength, shrinkage behavior, and resistance to fatigue, impact, and thermal shock [23,24]. Nowadays, various fibers, including polypropylene fibers (PPs) [25,26,27], polyamide fibers (PAs), polyvinyl alcohol fibers (PVAs) [28,29,30], steel fibers (STs) [31,32,33], and glass (GL) fibers [34,35], have been widely used, each offering potential benefits or drawbacks to concrete properties. Lee et al. [36] investigated slag-based alkali-activated mortar reinforced geopolymers with oiled PVAs and demonstrated the feasibility of achieving a tensile strain of up to 4.7%. Natali et al. [37] modified some properties of alkali-activated ladle-slag at 7 d by employing PVAs, and the enhancement in ductility after the first crack load was obvious. Zhang et al. [38,39] investigated the behavior of the short PVA-reinforced fly ash-metakaolin geopolymer boards at 28 d. Their conclusion demonstrated that the incorporation of high-volume PVAs changed the impact failure mode of geopolymer boards from a brittle to ductile pattern. Feng Hu et al. [40]. utilized fly ash and PEs in the preparation of engineered geopolymer composites (EGCs), and the resulting material has been effectively implemented in the construction of subway tunnel shield segments. Yuan et al. [41]. investigated Yellow River sand (YRS) in engineered cementitious composites (ECCs), finding that 100% YRS substitution enhances strength, with the compressive strength and flexural strength increasing by 26.4 MPa and 11.5 MPa, respectively, while 75% substitution provides optimal ductility, promoting sustainable concrete applications. Akturk et al. [42] demonstrated that steel fiber or polypropylene fiber addition improved the flexural strength of sodium carbonate-activated slag mortars. Zhou et al. [43] showed that increasing basalt fiber dosage enhanced the flexural and splitting tensile strength of fiber-reinforced alkali-activated slag concrete. Abdollahnejad et al. [44] incorporated various fiber types into alkali-activated slag mortar, finding improvements in flexural strength, while the effect on compressive strength varied depending on the fiber type.

The Yellow River sediment significantly differs from conventional river sand used in construction materials in terms of particle gradation and physicochemical properties. The sediment particles are predominantly fine, falling within the silt and clay range (<0.075 mm), with shapes that are mostly flaky or angular, featuring rough surfaces and a large specific surface area. Additionally, the sediment has a high mud content (typically exceeding 10%). These characteristics result in notable differences in the performance of geopolymer materials prepared using Yellow River sediment as an aggregate compared to those using conventional river sand. Consequently, the effects of fibers on the performance of Yellow River sediment-based geopolymer ecological cementitious materials cannot be entirely extrapolated from existing studies. Targeted adaptive research and exploration are necessary to understand and optimize their performance. However, there is limited research on the effects of various fibers on the properties of Yellow River sediment geopolymers and the underlying mechanisms.

Based on the aforementioned background, this paper selects steel fibers (hereinafter referred to as STs), polyvinyl alcohol fibers (hereinafter referred to as PVAs), and polyethylene fibers (hereinafter referred to as PEs) to systematically investigate the influence of different fiber types and dosages on the workability and strength of slag–Yellow River sediment geopolymer ecological cementitious materials by setting varying dosages. Combined with micro-testing methods such as TG-DTA, XRD, and SEM-EDS, the influence mechanisms of STs, PVAs, and PEs on the characteristic products and microstructure of slag–Yellow River sediment geopolymer ecological cementitious materials will be explored. The study aims to provide valuable insights into the engineering applications of fiber-reinforced slag–Yellow River sediment geopolymer materials.

## 2. Materials and Methods

### 2.1. Raw Material and Mixed Proportion

#### 2.1.1. Raw Material

**Figure 1:**
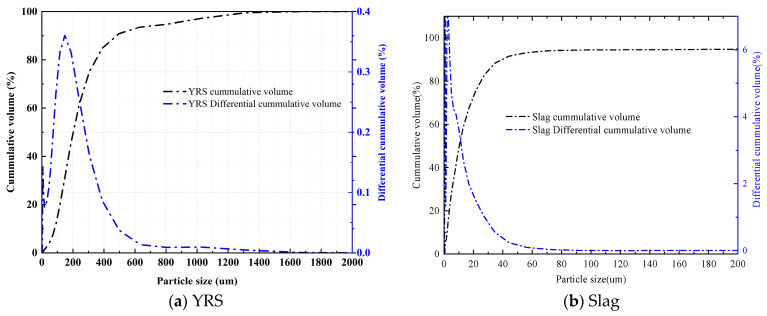
Particle size distribution curves of YRS and slag.

**Figure 2:**
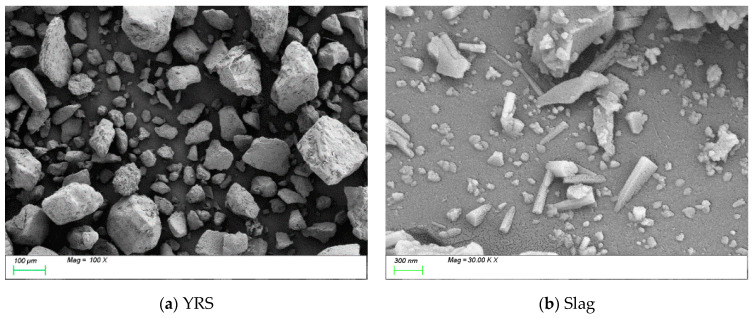
SEM images of YRS and slag.

The raw materials used in the experiment include Yellow River sediment (Abbreviate as YRS), slag, hybrid fiber, NaOH, and water glass. The Yellow River sediment was collected from the Xixiayuan Reservoir in Henan Province, initially in a moist state, and then dried before being used as a raw material. The chemical composition and particle size analysis of the YRS and slag were conducted using XRF and the Malvern Mastersizer-2000 laser particle size analyzer (UK). The results are shown in Table 1 and Figure 1, respectively. The slag used in this experiment is of grade S105, with a median particle size of 10.28 μm, a hydraulic coefficient of 2.17, an activity coefficient of 0.48, and an alkaline coefficient of 1.14. SEM images, shown in Figure 2, revealed that YRS particles displayed irregular geometric shapes, with considerable variation in particle size. Slag particles demonstrated a heterogeneous morphology, consisting of irregular shapes, flakes, spheres, and angular fragments. Three hybrid fibers were selected, namely short and fine copper-plated steel fibers, short-cut PVAs, and PEs, the specific performance indicators of which are shown in Table 2. Water glass, commonly known as sodium silicate, is a general term for Na_2_O·nSiO_2_, where n is usually referred to as the modulus. It is a transparent glassy solution composed of alkali metal silicates. The chemical composition of water glass essentially depends on the molecular ratio n between SiO_2_ and alkali metal oxides (Na_2_O or K_2_O), known as the water glass modulus. In this experiment, liquid sodium silicate produced by Shandong Yourui Chemical Co., Ltd. (Zibo, China) was used, with the physicochemical parameters shown in Table 3. Pure NaOH was used to adjust the water glass molar ratio to 1.2, and the Na_2_O content of the binder was set to 5%. Tap water was used for mixing in the experiment. The modulus adjustment equation is shown in Formula (1), which follows:

(1) $$Na_{2}O\cdot 2.18SiO_{2}+NaOH\to Na_{2}O\cdot nSiO_{2}+H_{2}O$$

#### 2.1.2. Mixed Proportion

Prior to this research, experimental investigations were conducted to determine the optimal mix proportion parameters, as detailed in Section 3.1 “Main mix proportion parameters determination”. Table 4 shows the mix proportion of slag–Yellow River sediment geopolymers with different fiber content. Before the experiment, the pre-weighed NaOH was thoroughly amalgamated with the sodium silicate. The mixture was subsequently concocted utilizing a uniaxial horizontal concrete mixer. Initially, the YRS along with the mineral admixtures were introduced into the mixer and agitated for a duration of 180 s. Following this, the fibers were incorporated into the mixer and the stirring continued for an additional 240 s. Subsequently, water was introduced into the mixer and the mixture was stirred for a further 180 s. Thereafter, the water glass was added and the mixture was stirred for 120 s. Immediately following this, the mixture was promptly poured into molds and then subjected to consolidation on a vibration table for a period ranging between 60 and 90 s. After 24 h of curing at room temperature, the specimens were demolded and stored in a curing chamber at 20 ± 2 °C and 95 ± 5% relative humidity until the designed curing ages [45,46].

### 2.2. Experimental Method

In this experiment, the effects of ST, PVA, and PE contents on the workability, mechanical elements, and characteristics of these products, alongside the microstructural properties of Yellow River sediment ecological cementitious materials, were analyzed through their compressive and splitting tensile strength, thermos gravimetric analysis, X-ray diffraction (XRD) analysis, and a scanning electron microscopy (SEM) test. Table 5 shows the grouping of the test, including the size and number of each test and specimen.

#### 2.2.1. Workability Test

The workability is a key index against which to measure the mixture. Whether it is easy to transport, pour, vibrate, and form, plays an extremely important role in ensuring the construction quality. Slump and expansion are two important evaluation indexes to measure the workability of mixtures. In this study, a slump bucket with a diameter of 50 mm at the top and 100 mm at the bottom and a height of 150 mm and a 60 cm × 60 cm flat plate were used to test the slump and slump flow of the mixture.

#### 2.2.2. Strength Test

In order to fully evaluate the effect of fiber on the strength and toughness of matrix, the cube specimens with a side length of 100mm were used to test their compressive strength and splitting tensile strength, following the requirements outlined in GB/T 50081-2019 “Standard for Test Methods of Physical and Mechanical Properties of Concrete” [47]. The tension–compression ratio was calculated according to the test results.

#### 2.2.3. X-Ray Diffraction Analysis

Using a Japanese Physical X-ray diffractometer, the sample was immersed in anhydrous ethanol for more than 7 days to terminate the reaction, then taken out of the sample and put it into a vacuum-drying oven for drying. After drying, the sample was ground, passed through a 200-mesh sieve, and put into a glass groove for testing. The sampling interval was 0.04° (2θ), the sampling speed was 1°/min, and the scanning angle range was 5°–70° (2θ).

#### 2.2.4. Thermos Gravimetric Analysis

A ZCT-B simultaneous thermal analyzer (BEIJING JINGYI HITECHINSTRUMENT Co., Ltd., Beijing, China) was employed and the sample preparation method was the same as XRD. About 20mg of the sample was taken to be tested. The heating range was 30 °C~1000 °C, the heating rate was controlled at 10 °C/min, and the heating atmosphere was argon gas. The DTA curves for each group of samples were obtained.

#### 2.2.5. Scanning Electron Microscopy Test

The microstructure of the cement paste samples was observed using a Sigma 300 field emission environmental scanning electron microscope (Carl Zeiss AG, Oberkochen, Germany). After curing to 28 d old, the samples were broken with pliers, immersed in anhydrous ethanol for more than 7 days to terminate the reaction, then place in a vacuum-drying oven for drying, and the test was conducted after the drying process was complete.

## 3. Experiment Results and Analysis

### 3.1. Main Mix Proportion Parameters Determination

**Figure 3:**
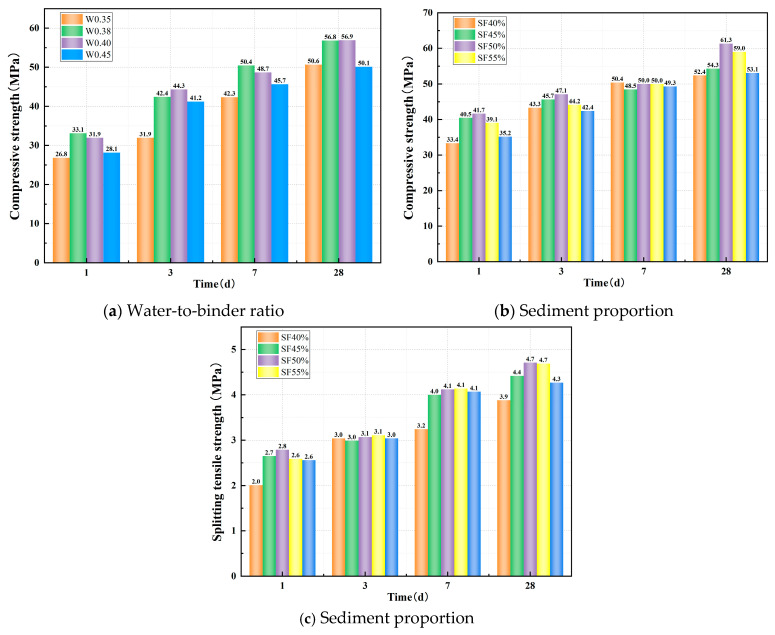
Effect of main mix proportion parameters on the strength of a slag–Yellow River sediment geopolymer.

Before conducting this study, it was essential to determine the appropriate mix proportion parameters. The water-to-binder ratio is a critical factor influencing the compressive strength of the matrix. An excessively low water-to-binder ratio can result in an overly concentrated alkali activator, a rapid reaction kinetics, and an uneven distribution of reaction products, which can ultimately degrade the material’s mechanical performance. Conversely, an excessively high water-to-binder ratio would dilute the alkali activator, slow down the reaction rate (leading to insufficient product formation), and promote the evaporation of free water, which would increase porosity and reduce the compressive strength of the material. Consequently, this study first evaluated the effect of the water-to-binder ratio on the compressive strength of a slag–Yellow River sediment geopolymer. It should be noted that the total water content comprises two components: the water in the sodium silicate solution and the additional water added to the mixture. As shown in Figure 3a, the compressive strength of the matrix does not simply increase with a decreasing water-to-binder ratio; rather, it exhibits an optimal range of 0.38–0.40. Considering the subsequent need to incorporate fibers while maintaining the required workability, a water-to-binder ratio of 0.40 was selected for this study.

Additionally, since the sediment from the Xixiayuan Reservoir used in this study has relatively fine particle sizes (with a most probable particle size of approximately 0.15 mm), its particle gradation and physicochemical composition differ significantly from conventional sand. Coupled with higher clay content, the influence of sediment proportion on matrix strength cannot be overlooked. A low proportion of Yellow River sediment offers limited economic benefits, while an excessively high proportion can lead to significant defects within the matrix. Furthermore, the clay fines tend to absorb a large amount of free water, which adversely affects the workability of the mixture. For these reasons, this study set the sediment proportion range at 40–60% and investigated its impact on matrix strength. As shown in Figure 3b,c, the matrix strength initially increases and then decreases as the sediment content rises. Both compressive strength and splitting tensile strength reach relatively high levels at a sediment proportion of 50%. Therefore, this study ultimately identified the optimal parameters as a water-to-binder ratio of 0.38 and a sediment proportion of 50%.

### 3.2. Workability

**Figure 4:**
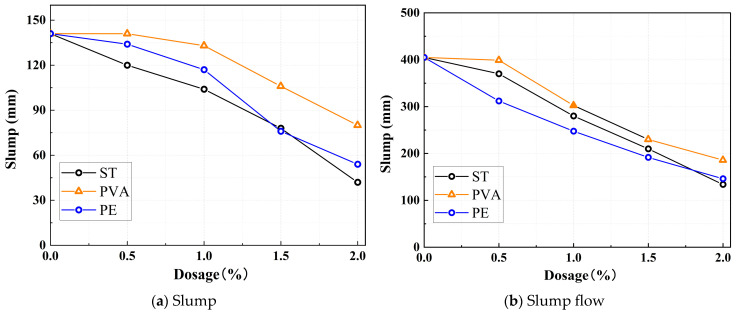
Effect of fiber on workability for (**a**) slump, and (**b**) slump flow.

Figure 4 illustrates the effects of different mineral admixture replacement rates on the slump and slump flow of the mixture. Figure 4a specifically shows the influence of mineral admixture replacement rates on the slump. As observed in the figure, different mineral admixtures exhibit distinct effects on slump. With increasing steel fiber content, the slump of the mixture decreases significantly. When the steel fiber content reaches 2%, the slump drops sharply from 141 mm to 42 mm. PVAs and PEs display similar two-stage variation patterns. When the fiber content is within 1%, their impact on the fluidity of the paste is minimal. However, at a fiber content of 1%, the slumps for PVA-1% and PE-1% are 133 mm and 117 mm, respectively. Further increases in fiber content led to a marked decline in slump; at 2% fiber content, the slumps for PVA-2% and PE-2% plummet to 80 mm and 54 mm. Unlike slump, the slump-flow decreases significantly with increasing fiber content. As shown in Figure 4b, when the fiber content reaches 2%, the slump-flow values for STs, PVAs, and PEs drop sharply to 134 mm, 186 mm, and 146 mm, respectively.

This phenomenon occurs because steel fibers, due to their high density, increase the self-weight of the mixture. Additionally, their elevated surface roughness intensifies internal friction within the paste, leading to significant reductions in both slump and slump flow. In contrast, organic fibers, being lightweight and flexible, exhibit minimal impact on slump at low incorporation levels. However, their inherent water-absorbing properties deplete free water in the mixture, thereby reducing slump flow. When the fiber content exceeds a critical threshold, organic fibers form three-dimensional networks and agglomerates, which impede the flow of the paste, resulting in continuous declines in both slump and slump flow. In comparison, steel fibers exert the most pronounced adverse effects on workability. Among the organic fibers, PEs exhibit a more significant impact on performance degradation than PVAs.

### 3.3. Strength

#### 3.3.1. Compressive Strength

**Figure 5:**
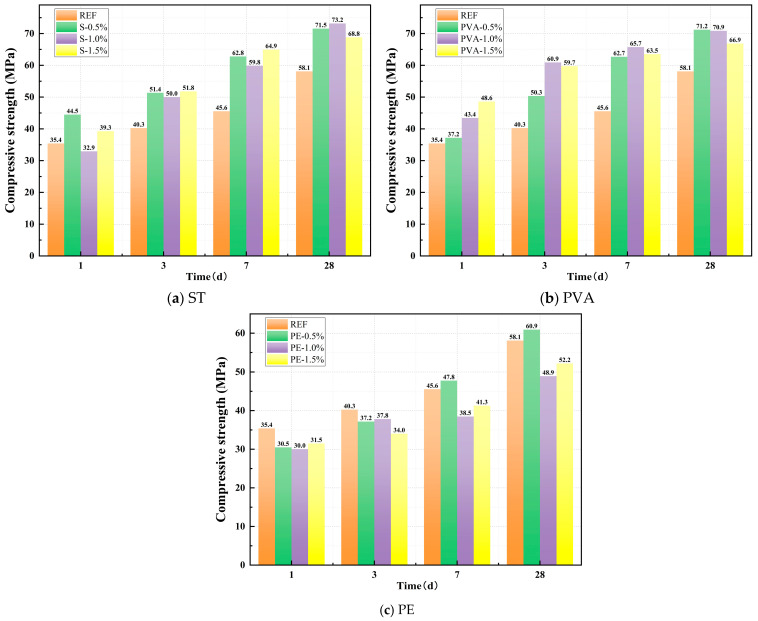
Effect of fiber on compressive strength.

Figure 5a–c illustrates the effects of STs, PVAs, and PEs on the compressive strength of the matrix. As shown in Figure 5a, the incorporation of an appropriate dosage of steel fibers significantly enhances the compressive strength of the matrix. After 1 day of curing, the compressive strength of the control group (without fibers) was 35.4 MPa. For ST0.5, ST1.0, and ST1.5, the compressive strengths were 44.5 MPa, 32.9 MPa, and 39.3 MPa, respectively. Except for ST1.0, which was slightly lower than the control group, ST0.5 and ST1.5 exhibited compressive strength increases of 25.68% and 10.93%, respectively, compared to the control group. As curing continued, all steel fiber-incorporated mixes demonstrated significantly higher compressive strengths than the control group. After 28 days of standard curing, the control group reached a compressive strength of 58.1 MPa, while ST0.5, ST1.0, and ST1.5 achieved compressive strengths of 71.5 MPa, 73.2 MPa, and 68.8 MPa, respectively, representing increases of 23.11%, 25.93%, and 18.43% over the control group.

From Figure 5b, it can be observed that the incorporation of PVAs also significantly enhances the compressive strength of the matrix. After 1 day of curing, the compressive strengths of PVA0.5, PVA1.0, and PVA1.5 were 37.2 MPa, 43.4 MPa, and 48.6 MPa, respectively, representing increases of 5.06%, 22.71%, and 37.29% compared to the control group. As the curing age increased, all PVA-incorporated mixes maintained significantly higher compressive strengths than the control group. After 28 days of standard curing, the compressive strengths of PVA0.5, PVA1.0, and PVA1.5 reached 71.2 MPa, 70.9 MPa, and 66.9 MPa, respectively, corresponding to increases of 22.53%, 21.96%, and 15.06% over the control group.

From Figure 5c, it can be observed that, unlike steel fibers and PVAs, incorporating PEs slightly reduces the early-age compressive strength of the matrix. At 1 day of curing, the compressive strengths of PE0.5, PE1.0, and PE1.5 were 30.5 MPa, 30.0 MPa, and 31.5 MPa, respectively, representing decreases of 13.90%, 15.15%, and 11.05% compared to the control group. As the curing age continued to increase, the strength growth effect gradually became apparent under appropriate dosage ranges. After 28 days of standard curing, the compressive strengths of PE0.5, PE1.0, and PE1.5 reached 60.9 MPa, 48.9 MPa, and 52.2 MPa, respectively. While PE0.5 exhibited a 4.85% increase over the control group, PE1.0 and PE1.5 showed reductions of 15.81% and 10.25%, respectively.

In summary, both steel fibers and PVAs significantly enhance the early-age and later-age compressive strength of the control group, albeit through distinct mechanisms. Steel fibers primarily enhance compressive performance due to their high stiffness and tensile strength, while PVAs mitigate crack propagation through their flexibility and strong bonding properties. In contrast, the incorporation of PEs results in a slight reduction in early-age compressive strength for the sodium silicate-slag powder alkali-activated cementitious material. However, within appropriate dosage ranges, a gradual strength gain is observed at later ages, although the effectiveness of PEs remains less pronounced when compared to STs and PVAs.

#### 3.3.2. Splitting Tensile Strength

**Figure 6:**
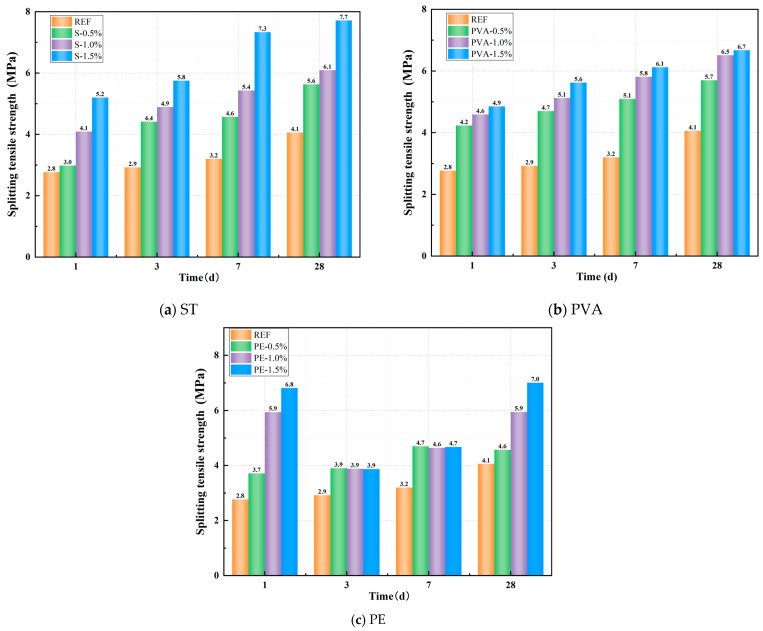
Effect of fiber on splitting tensile strength.

Figure 6a–c illustrates the effects of STs, PVAs, and PEs on the compressive strength of geopolymer paste. From Figure 6a, it can be observed that incorporating steel fibers significantly enhances the splitting tensile strength of the matrix, with the effect becoming more pronounced as the fiber dosage increases. At 1 day of curing, the splitting tensile strength of the control group (without fibers) was 2.77 MPa. For ST0.5, ST1.0, and ST1.5, the splitting tensile strengths were 2.98MPa, 4.09MPa, and 5.20MPa, respectively, representing increases of 7.58%, 47.65%, and 87.73% compared to the control group. As the curing age increased, all steel fiber-incorporated mixes exhibited continuous growth in splitting tensile strength, consistently outperforming the control group. After 28 days of standard curing, the control group reached a splitting tensile strength of 4.06 MPa, while ST0.5, ST1.0, and ST1.5 achieved 5.63 MPa, 6.09 MPa, and 7.71 MPa, respectively, corresponding to improvements of 38.67%, 50.00%, and 89.90% over the control group.

From Figure 6b, it can be observed that incorporating PVAs also significantly improves the splitting tensile strength of the matrix. At 1 day of curing, the splitting tensile strengths of PVA0.5, PVA1.0, and PVA1.5 were 4.23 MPa, 4.59 MPa, and 4.85 MPa, respectively, representing increases of 52.71%, 65.70%, and 75.09% compared to the control group. As the curing age increased, all PVA-incorporated mixes maintained significantly higher splitting tensile strengths than the control group. After 28 days of standard curing, the splitting tensile strengths of PVA0.5, PVA1.0, and PVA1.5 reached 5.70 MPa, 6.51 MPa, and 6.67 MPa, respectively, corresponding to improvements of 40.39%, 60.34%, and 64.29% over the control group.

From Figure 6c, it can be observed that, unlike its effect on compressive strength, the incorporation of PEs significantly enhances the splitting tensile strength of the matrix. After 1 day of curing, the splitting tensile strengths of PE0.5, PE1.0, and PE1.5 were 3.71 MPa, 5.94 MPa, and 6.82 MPa, respectively, representing increases of 34.09%, 114.51%, and 146.08% compared to the control group. As the curing age progressed, the rate of splitting tensile strength growth in PE-incorporated mixes slowed, yet their strengths remained higher than the control group. After 28 days of standard curing, the splitting tensile strengths of PE0.5, PE1.0, and PE1.5 reached 4.57 MPa, 5.94 MPa, and 7.01 MPa, respectively, corresponding to improvements of 12.61%, 46.42%, and 72.61% over the control group. In summary, steel fibers, PVAs, and PEs all significantly enhance the tensile strength of the control group, with the toughening effect becoming more pronounced as the fiber dosage increases. Comparatively, PEs demonstrate the most significant enhancement in early-age tensile performance, while STs and PVAs exhibit more sustained and balanced strength improvements across all curing ages.

#### 3.3.3. Tension–Compression Ratio

The tension–compression ratio is a critical parameter for evaluating the brittle characteristics of concrete, reflecting the material’s mechanical behavior under both tensile and compressive stress conditions. A lower tension–compression ratio indicates that concrete is more susceptible to brittle failure under tensile stress, exhibiting poor crack resistance. In contrast, a higher tension–compression ratio suggests improved material toughness, which helps control crack formation and delays crack propagation, thereby enhancing the overall performance of concrete structures. Variations in the tension–compression ratio not only influence the load-bearing capacity of structures but also have significant implications for their durability and service life. Therefore, optimizing the tension–compression ratio is essential for ensuring structural integrity and long-term durability in engineering applications.

**Figure 7:**
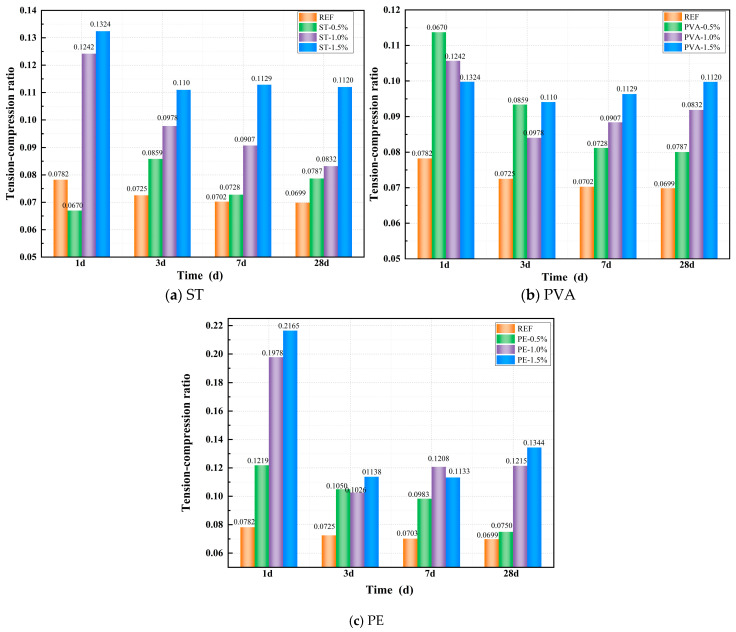
Effect of fiber on tension–compression ratio.

Figure 7a–c illustrates the effects of STs, PVAs, and PEs on the tension–compression ratio of a geopolymer paste. From Figure 7a, it can be observed that all PVA-incorporated mixes exhibit significantly higher tension–compression ratios than the control group, with the ratio increasing more markedly as fiber dosage rises. At 1 day of curing, the tension–compression ratio of ST0.5 was 14.40% lower than the control group, while ST1.0 and ST1.5 showed increases of 58.73% and 69.23%, respectively. As the curing age increased, all steel fiber-incorporated mixes maintained significantly higher tension–compression ratios than the control group. After 28 days of standard curing, the tension–compression ratios of ST0.5, ST1.0, and ST1.5 increased by 12.64%, 19.11%, and 60.35%, respectively, compared to the control group.

From Figure 7b, it can be observed that all PVA-incorporated mixes exhibit significantly higher tension–compression ratios than the control group. However, unlike the trend observed with steel fibers, the early-age tension–compression ratio decreases as fiber content increases. After 1 day of curing, the tension–compression ratios of PVA0.5, PVA1.0, and PVA1.5 were 45.36%, 35.04%, and 27.53% higher than the control group, respectively. As the curing age progressed, the influence of higher fiber content on the tension–compression ratio became more pronounced. After 28 days of standard curing, the tension–compression ratios of PVA0.5, PVA1.0, and PVA1.5 increased by 14.58%, 31.48%, and 42.79%, respectively, compared to the control group.

From Figure 7c, it can be observed that all PE-incorporated mixes exhibit significantly higher tension–compression ratios than the control group, with the ratio increasing more markedly as fiber dosage rises. At 1 day of curing, the tension–compression ratios of PE0.5, PE1.0, and PE1.5 were 34.09%, 114.51%, and 146.08% higher than the control group, respectively. As the curing age progressed, the tension–compression ratio exhibited a declining trend due to the slower growth of splitting tensile strength and the continuous improvement in compressive strength in the PE-incorporated mixes. Nevertheless, at all curing ages, the tension–compression ratios of PE-incorporated specimens remained significantly higher than those of the control group. After 28 days of standard curing, the tension–compression ratios of PE0.5, PE1.0, and PE1.5 increased by 7.41%, 73.91%, and 92.32%, respectively, compared to the control group.

### 3.4. Five-Dimensional Evaluation

**Figure 8:**
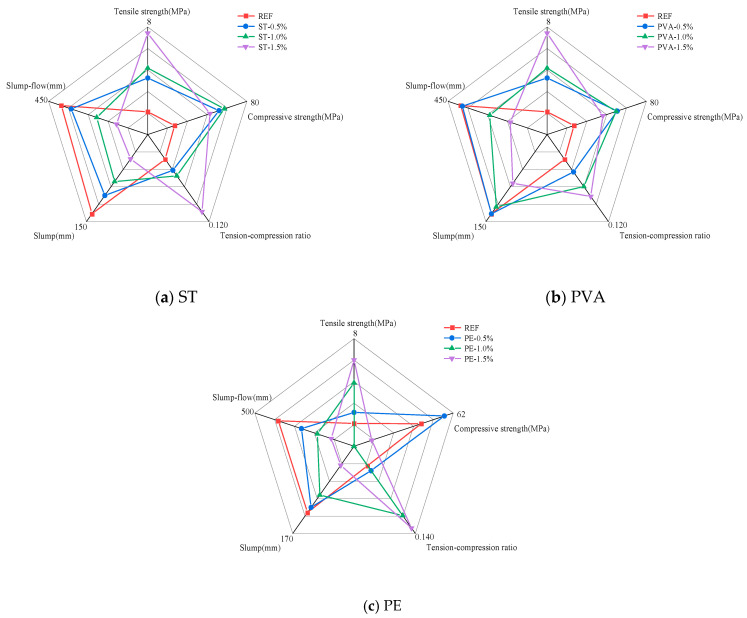
Five-dimensional evaluation diagram under different ST, PVA, and PE content.

Based on the experimental results, this study selected five key indicators—compressive strength, splitting tensile strength, tension–compression ratio, slump, and slump flow—and employed a five-dimensional evaluation method [48] for comparative analysis to determine the optimal dosage of STs, PVAs, and PEs. The multidimensional evaluation results of slag–Yellow River sediment geopolymers under different fiber dosages are shown in Figure 8. From Figure 8a, it can be observed that incorporating 1.0% steel fibers provides a balanced enhancement of matrix strength: compressive strength, splitting tensile strength, and tension–compression ratio increased by 25.93%, 50.00%, and 19.11%, respectively, compared to the control group. In contrast, 1.5% steel fibers yielded the most significant improvement in tensile performance, with increases of 18.43%, 89.90%, and 60.35% in compressive strength, splitting tensile strength, and tension–compression ratio, respectively. However, at this dosage (1.5%), workability declined significantly. From Figure 8b, similar to steel fibers, 1.0% of PVAs achieved balanced strength improvements as the compressive strength, splitting tensile strength, and tension–compression ratio increased by 21.96%, 60.34%, and 31.48%, respectively. Meanwhile, 1.5% PVAs maximized tensile strength enhancement, with increases of 15.06%, 64.29%, and 42.79% in the respective metrics. Nevertheless, workability also deteriorated markedly at this higher dosage. From Figure 8c, PEs exhibited uneven strength enhancement. Incorporating 0.5% PEs delivered the best compressive strength improvement but limited tensile performance gains, while 1.5% PEs maximized tensile strength enhancement but adversely affected workability and compressive strength. In summary, to ensure satisfactory workability and balance performance metrics, 1.0% dosage is recommended for both STs and PVAs.

## 4. Influence Mechanism Analysis

### 4.1. Reaction Products

**Figure 9:**
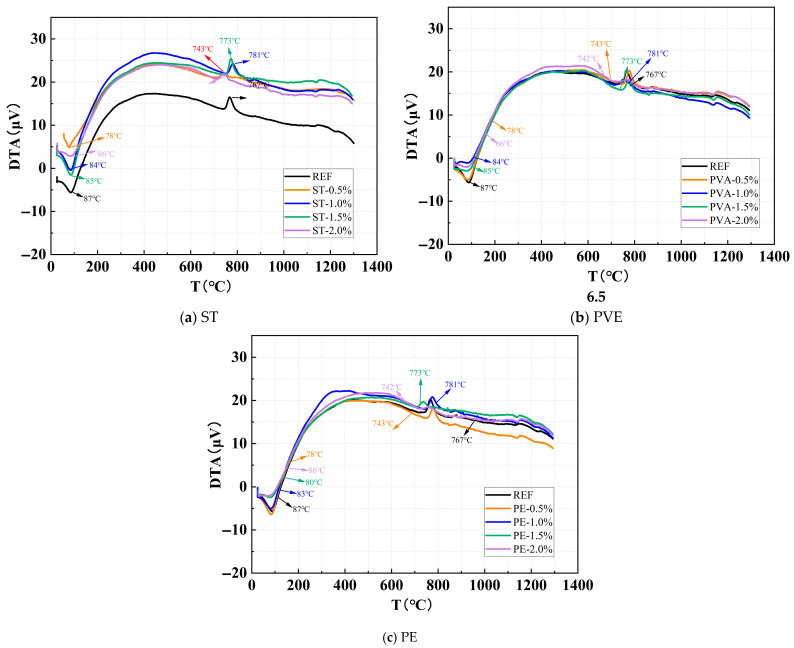
Comparative study of thermal analysis curves under different ST, PVA, and PE content.

Due to the complex composition of the silted sediment, paste samples excluding sediment were prepared for XRD and TGA tests to avoid interference from impurities and minerals in the sediment. Figure 9 shows the TGA curves under different ST, PVA, and PE contents. As observed in the figure, the TGA curves exhibit two primary thermal decomposition peaks: one in the 70–100 °C temperature range and another in the 700–850 °C range.

From Figure 9a, it can be observed that the low-temperature decomposition peaks of the control group, ST0.5, ST1.0, ST1.5, and ST2.0 are located at 87 °C, 78 °C, 84 °C, 85 °C, and 86 °C, respectively, while their high-temperature decomposition peaks are positioned at 767 °C, 743 °C, 781 °C, 773 °C, and 742 °C, respectively. When the steel fiber content reaches 2.0%, the intensity of the high-temperature decomposition peak decreases, indicating that excessive steel fibers reduce the number of characteristic gel products formed. However, the type of reaction products remains unchanged.

From Figure 9b, it can be observed that as the PVA content increases, the low-temperature decomposition peaks of the control group, PVA0.5, PVA1.0, PVA1.5, and PVA2.0 are located at 87 °C, 85 °C, 84 °C, 81 °C, and 79 °C, respectively, while their high-temperature decomposition peaks occur at 767 °C, 777 °C, 756 °C, 774 °C, and 777 °C, respectively. The high-temperature decomposition peak intensities for PVA1.5 and PVA2.0 are lower than those of other mixes, and the low-temperature decomposition peak intensities for PVA1.0, PVA1.5, and PVA2.0 are also reduced. These results indicate that while PVAs do not alter the types of reaction products, the hydroxyl (-OH) groups on PVAs impart remarkable hydrophilicity (with a typical contact angle of <30°) and they influence the distribution of free water and the quantity of characteristic gel products formed. Moreover, at equivalent dosages, the impact of PVAs on these properties is more pronounced compared to steel fibers.

From Figure 9c, it can be observed that as the PE content increases, the low-temperature decomposition peaks of the control group, PE0.5, PE1.0, PE1.5, and PE2.0 are located at 87 °C, 84 °C, 83 °C, 80 °C, and 72 °C, respectively, while their high-temperature decomposition peaks occur at 767 °C, 778 °C, 775 °C, 736 °C, and 757 °C, respectively. Both the low-temperature and high-temperature decomposition peak intensities for PE1.5 and PE2.0 are lower than those of other mixes. These results indicate that while PEs do not alter the types of reaction products, they significantly influence the distribution of free water and the quantity of characteristic gel products formed. Furthermore, at equivalent dosages, the impact of PEs on these properties is more pronounced compared to steel fibers.

In summary, the incorporation of steel fibers, PVAs, and PEs does not alter the types of characteristic reaction products in alkali-activated cementitious materials. However, these fibers significantly influence the formation of gel-like products and the distribution of free water, thereby impacting both the quantity and the thermal decomposition behavior of the reaction products. The effects of PVAs and PEs are notably more pronounced compared to steel fibers, highlighting their more substantial influence on the microstructural properties of the matrix.

**Figure 10:**
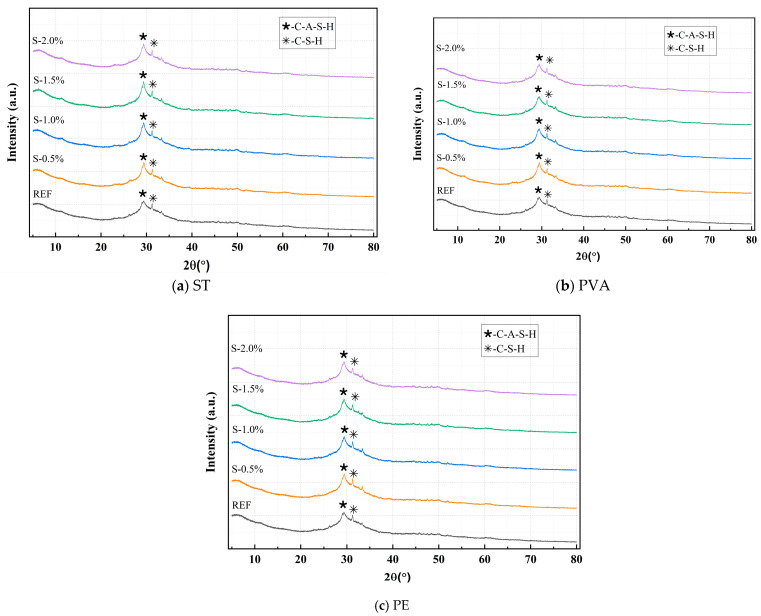
Comparative study of XRD patterns under different ST, PVA, and PE content.

Figure 10 shows the XRD patterns of a matrix under different ST, PVA, and PE content. Respectively. As can be seen from the figures, whether in Figure 10a,b or Figure 10c, the main reaction products of the sample are C-A-S-H. The results indicate that fibers influence the matrix product content without inducing the formation of new reaction products. As fiber content increases, the peak intensity of the C-A-S-H and C-S-H phases decreases. While all fiber types affect the formation of gel-like products, their mechanisms of action vary. With increasing steel fiber content, excessive fiber aggregation within the matrix leads to the formation of additional interfacial transition zones (ITZs), which disrupt the spatial distribution of the reactions, resulting in the non-uniform dispersion of primary gel-like products and incomplete polymerization. In contrast, PVAs and PEs influence the reactions through distinct mechanisms. PVAs, due to their hydrophilic nature, adsorb free water, reducing the available water for the “dissolution-depolymerization-condensation” reaction and thereby limiting the formation of characteristic gel products. Additionally, PVAs alter the pore structure of the matrix, further modifying the spatial distribution of the reactions. Conversely, PEs, owing to their hydrophobicity, impede water migration and distribution, disrupting the homogeneity of the reaction and consequently reducing the quantity of gel products formed. These observations align with thermal analysis results which indicate that fibers primarily act as physical reinforcements within the system. Their chemically inert nature ensures minimal interaction with the alkali-activation reaction process. As a result, fibers influence reaction uniformity and the quantity of reaction products formed primarily through physical mechanisms, without altering the types of reaction products.

### 4.2. Matrix Microstructure

**Figure 11:**
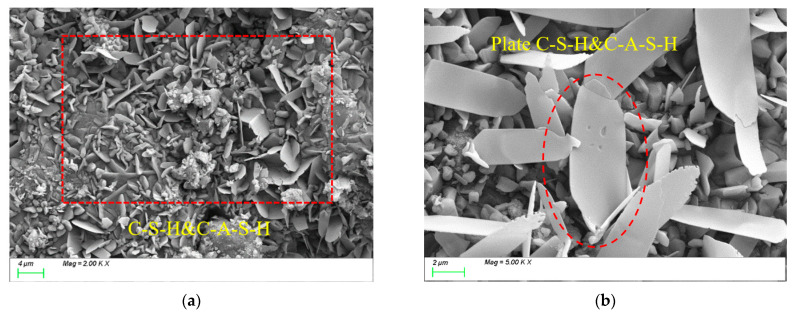
Microscopic image of REF. (**a**) Microstructure, (**b**) characteristic product.

Figure 11 presents the SEM test results of the control group. As shown in Figure 11a, after 28 days of curing, numerous plate-like C-(A)-S-H gels were formed within the matrix. These gel products exhibited sufficient growth [49] and effectively filled the matrix voids, forming a uniform, dense, continuous, and intact microstructure, thereby contributing to the high compressive strength. From Figure 11b, it can be observed that most slag particles were completely reacted, with tightly packed, well-developed gel products displaying a higher degree of polymerization. This resulted in a dense matrix structure [50]. This phenomenon is attributed to the appropriate alkali content, which enhances the dissolution rate of slag particles, accelerates the reaction process, increases the reaction extent, and ultimately promotes the formation of C-A-S-H and C-S-H gels.

**Figure 12:**
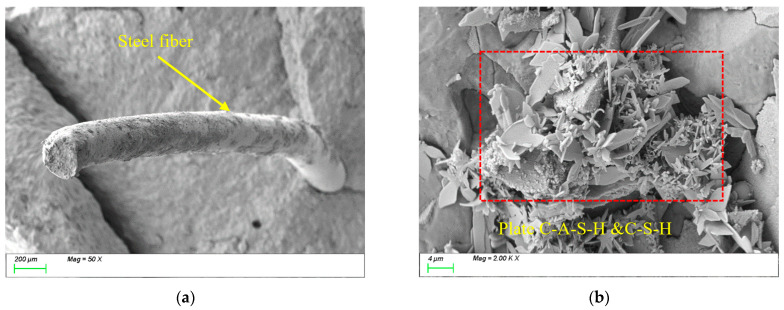
Microscopic image of S0.5. (**a**) Steel fiber distribution, (**b**) characteristic product.

Figure 12 presents the SEM results of the sample incorporating 0.5% STs. As shown in Figure 12a, the steel fibers exhibit tight physical bonding with the matrix structure, with a distinct interfacial transition zone characterized by strong mechanical interlocking. The high surface roughness of the steel fibers enhances mechanical anchorage with the matrix, while their high modulus, stiffness, and large diameter effectively improve the strength and toughness of the matrix. From Figure 12b, it can be observed that the primary reaction products within the matrix remain plate-like C-(A)-S-H gels, indicating that the incorporation of steel fibers does not alter the types or microstructural morphology of the characteristic reaction products. However, the high modulus of steel fibers enhances the material’s overall mechanical performance by inhibiting crack propagation through fiber-bridging effects.

**Figure 13:**
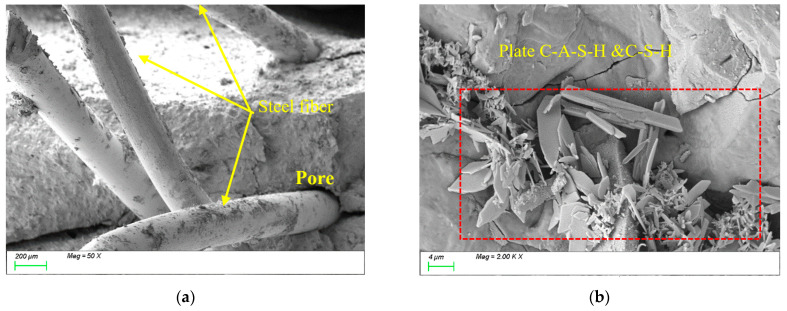
Microscopic image of S1.5. (**a**) Steel fiber distribution, (**b**) characteristic product.

Figure 13 presents the SEM results of the sample incorporating 1.5% STs. As shown in Figure 13a, the randomly oriented steel fibers form tightly interlocked networks, effectively enhancing the tensile performance of the matrix. However, the non-uniform distribution of fibers at this high dosage introduces voids at ITZs, compromising the matrix compactness. Consequently, compared to the 0.5% STs sample, the 1.5% STs specimen exhibits higher tensile strength but reduced compressive strength due to increased interfacial defects. From Figure 13b, the dominant reaction products within the matrix remain plate-like C-(A)-S-H gels, confirming that steel fiber dosage does not alter the types or microstructural morphology of characteristic products. Nevertheless, the voids and defects at ITZs formed by excessive steel fibers may act as stress concentration points, adversely affecting the material’s overall performance.

**Figure 14:**
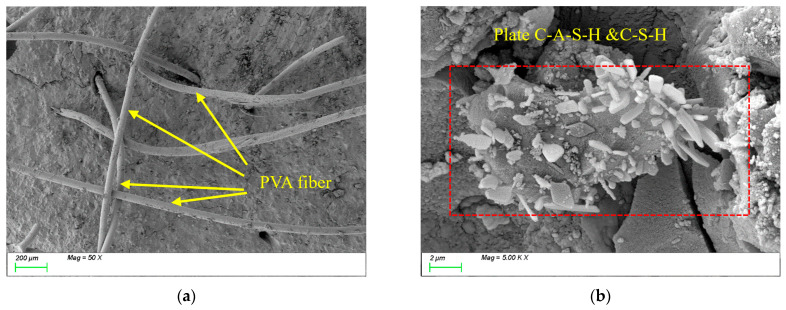
Microscopic image of PVA0.5. (**a**) Polyvinyl alcohol fiber distribution, (**b**) characteristic product.

Figure 14 presents the SEM results of the sample incorporating 0.5% PVAs. As shown in Figure 14a, the randomly oriented PVAs interconnect within the matrix, forming a three-dimensional spatial network structure. The flexibility and high ductility of PVAs enable them to effectively bridge microcracks at the microscale, significantly enhancing the material’s toughness. From Figure 14b, it can be observed that the internal matrix products are dominated by C-(A)-S-H gels, indicating that the incorporation of PVAs does not alter the types or microstructural morphology of characteristic reaction products. However, through fiber bridging effects, they suppress crack propagation, thereby improving the overall mechanical performance of the material.

**Figure 15:**
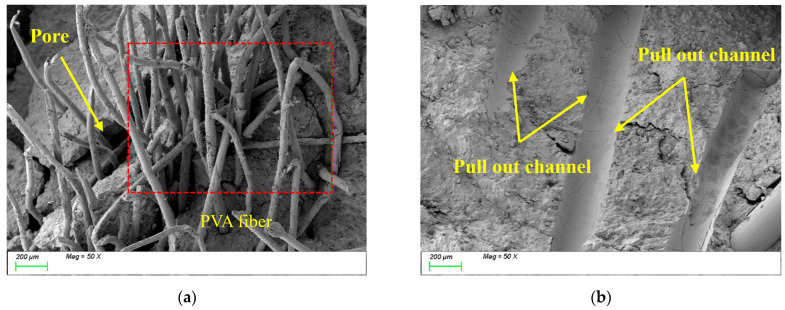
Microscopic image of PVA1.5. (**a**) Polyvinyl alcohol fiber distribution, (**b**) channels formed during fiber stretching.

Figure 15 presents the SEM results of the sample incorporating 1.5% PVAs. As shown in Figure 15a, the increased PVA content results in a dense network of randomly distributed, closely interlaced fibers, significantly enhancing the tensile performance of the matrix. During fiber stretching, channels formed within the matrix (as shown in Figure 15b), which consumed substantial energy and contributed to the development of the matrix’s integrity and tensile strength. However, the non-uniform distribution of high PVAs content introduces voids at ITZs (as shown in Figure 15a), reducing the matrix compactness and thereby adversely affecting compressive strength. In summary, the incorporation of PVAs markedly improves the toughness and tensile performance of sodium silicate-slag powder alkali-activated cementitious materials. Nevertheless, the fiber dosage must be optimized to avoid interfacial defects that compromise the material’s compactness and compressive strength.

**Figure 16:**
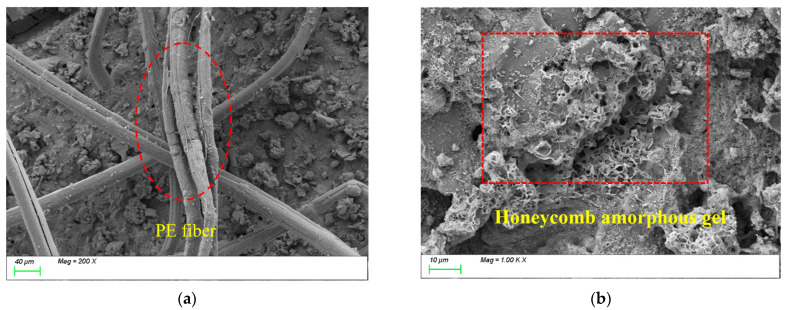
Microscopic image of PE0.5. (**a**) Polyethylene fiber distribution, (**b**) characteristic product.

Figure 16 presents the SEM results of the sample incorporating 0.5% polyethylene fibers (PEs). As shown in Figure 16a, compared to steel fibers, PEs exhibit lower density and a higher number of fibers per unit volume, facilitating spatial structural interlocking within the matrix and providing a certain reinforcing effect. From Figure 16b, it can be observed that the C-S-H and C-A-S-H gels within the matrix display honeycomb-like growth with a lower degree of polymerization and a relatively loose structure. This is likely attributed to the hydrophobicity and low surface energy of PEs, which weaken interfacial bonding with the matrix and result in inferior distribution uniformity compared to PVAs. Additionally, the incorporation of PEs may impede water migration and distribution, compromising reaction homogeneity and thereby restricting the quantity, distribution, and polymerization degree of characteristic gel products.

**Figure 17:**
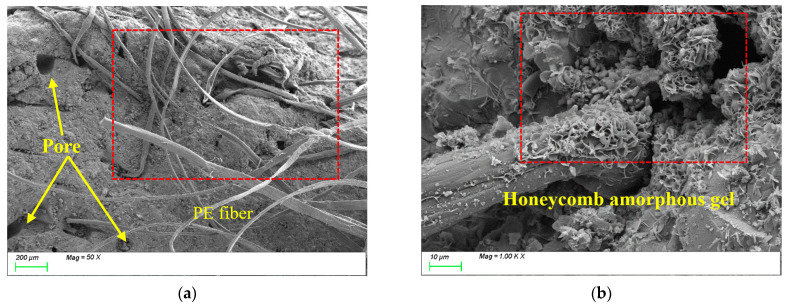
Microscopic image of PE1.5. (**a**) Polyethylene fiber distribution, (**b**) characteristic product.

Figure 17 presents the SEM results of the sample incorporating 1.5% polyethylene fibers (PEs). Compared to Figure 17a, the increased fiber content results in a significantly higher number of fibers within the matrix. The densely randomly oriented PEs form a three-dimensional network structure through extensive interlocking, which enhances the tensile strength of the matrix. However, the figure also reveals that excessive fiber content leads to non-uniform fiber distribution and the presence of large pores within the matrix, adversely affecting its compactness. From Figure 17b, it can be observed that the C-S-H and C-A-S-H gels within the matrix still exhibit honeycomb-like growth with a lower degree of polymerization and a loose gel structure. This results in weak bonding between the gels and PEs, which is likely one of the primary reasons why the compressive strength of the 1.5% PEs sample is lower than both the control group and the 0.5% PEs sample. In summary, the bridging effect of PEs primarily operates at the macroscopic scale. While high-tensile, high-elastic-modulus PEs improve the tensile performance of the matrix, their microscale reinforcement effect remains limited due to insufficient interfacial bonding and a compromised gel structure.

## 5. Conclusions

This study evaluated the impact of ST, PVA and PE on several critical properties of slag–Yellow River sediment geopolymers, including the workability, compressive strength, splitting tensile strength, characteristic products, and matrix microstructure. The following are the key findings of the study:

(1) As the fiber content increases, the workability (slump and spread) of the mixture significantly decreases. STs have the most pronounced effect on workability, with ST2.0 slump and spread reduced to 29.8% and 33.1% of the initial values, respectively. PVAs and PEs have a minor impact on slump at low dosages but significantly reduce workability when the dosage exceeds 1.0%. PVA2.0 slump and spread decrease to 56.7% and 45.9% of the initial values, respectively, while PE2.0 slump and spread decrease to 38.3% and 36.0% of the initial values, respectively. The addition of fibers increases internal friction and self-weight, with the high density of STs further hindering paste fluidity.
(2) An appropriate amount of STs and PVAs can significantly enhance the compressive strength and splitting tensile strength of the matrix. The 28-day compressive strength of ST1.0 and PVA1.0 increased by 25.93% and 21.96%, respectively, compared to the control group. The 28-day splitting tensile strength of ST1.5 and PVA1.5 increased by 89.90% and 64.29%, respectively, compared to the control group. The 28-day tensile-to-compressive ratio of ST1.5 and PVA1.5 increased by 60.35% and 42.79%, respectively, compared to the control group. In contrast, PEs primarily contribute to toughness, with PE1.5′s 28-day splitting tensile strength increasing by 72.61% and the 28-day tensile-to-compressive ratio increasing by 92.32% compared to the control group.
(3) With an increase in age, all fiber-reinforced specimens (containing STs, PVAs or PEs) exhibited significantly higher increasing rates in the tensile-to-compressive strength ratio compared to the reference group, and the ratio enhancement became more pronounced with higher fiber content; after 28 days of standard curing the ST-0.5, ST-1.0, and ST-1.5 mixtures showed 12.64%, 19.11%, and 60.35% improvements in the ratio, respectively, while the PVA-0.5, PVA-1.0, and PVA-1.5 mixtures achieved 14.58%, 31.48%, and 42.79% enhancements, respectively, and the PE-0.5, PE-1.0, and PE-1.5 mixtures attained 7.41%, 73.91%, and 92.32% increases, respectively, which conclusively verified the effective development of fiber bridging effects in the cementitious composites.
(4) STs, PVAs, and PEs do not alter the types of characteristic products in geopolymer cementitious materials, but they affect the generation of gel-like products and the distribution of free water to varying degrees. PVAs and PEs have a more significant impact than STs, and excessive fiber content can affect the generation of characteristic gel products and their thermal decomposition behavior.
(5) STs enhance the compressive performance of the matrix through their high modulus and stiffness and inhibit crack propagation through fiber bridging. PVAs suppress crack propagation through their flexibility and good bonding properties, while PEs improve the tensile performance of the matrix through their high tensile strength and elastic modulus. Although their reinforcing effect on the matrix at the microscopic scale is relatively weak, the interfacial properties between PEs and the cement matrix could be optimized through surface treatments (e.g., chemical modification or coating applications) in future studies, thereby enhancing fiber–matrix interactions.

## Figures and Tables

**Table 1:** Chemical compositions of YRS and slag (wt.%).

Minerals	SiO_2_	CaO	Al_2_O_3_	Fe_2_O_3_	K_2_O	TiO_2_	MgO	Other
YRS	68.64	8.40	12.33	3.25	2.55	0.74	2.05	2.04
slag	32.47	41.06	14.52	0.28	0.44	1.25	7.08	2.9

**Table 2:** Performance specification of fiber.

Fiber Type	Diameter (mm)	Density (g.cm^−3^)	Length (mm)	Tensile Strength (MPa)	Elasticity (GPa)	Elongation (%)
ST	0.22	7.9	13	2800	210	5
PVA	0.04	1.3	12	1560	41	6.5
PE	0.02~0.03	0.9	12	3378	125	3

**Table 3:** The chemical composition of sodium silicate.

SiO_2_/(%)	Na_2_O/(%)	H_2_O/(%)	Density/(g/cm^3^)	Modulus	Beaume
30	13.5	56.5	1.51	2.3	50

**Table 4:** Mix proportion for slag–Yellow River sediment geopolymers with different fiber content.

No.	Sand	NaOH	SS	Slag	ST	PVA	PE	Water
REF	1.000	0.020	0.128	0.660	—	—	—	0.192
ST-0.5%	1.000	0.020	0.128	0.660	0.032	—	—	0.192
ST-1.0%	1.000	0.020	0.128	0.660	0.065	—	—	0.192
ST-1.5%	1.000	0.020	0.128	0.660	0.097	—	—	0.192
PVA-0.5%	1.000	0.020	0.128	0.660	—	0.006	—	0.192
PVA-1.0%	1.000	0.020	0.128	0.660	—	0.011	—	0.192
PVA-1.5%	1.000	0.020	0.128	0.660	—	0.017	—	0.192
PE-0.5%	1.000	0.020	0.128	0.660	—	—	0.004	0.192
PE-1.0%	1.000	0.020	0.128	0.660	—	—	0.007	0.192
PE-1.5%	1.000	0.020	0.128	0.660	—	—	0.011	0.192

**Table 5:** Grouping of the workability, mechanical, and microstructural property tests.

Properties	Performance Index	Specimen Size	Quantity
Workability	Slump	—	—
	Slump flow	—	—
Strength	Compressive strength	100 mm × 100 mm × 100 mm	120
	Splitting tensile strength	100 mm × 100 mm × 100 mm	120
Characteristic products	Thermos gravimetric analysis	40 mm × 40 mm × 40 mm	30
	X-ray diffraction analysis	40 mm × 40 mm × 40 mm	30
Microstructural properties	Scanning electron microscopy	40 mm × 40 mm × 40 mm	30

## Data Availability

The original contributions presented in the study are included in the article. Further inquiries can be directed to the corresponding author.

## Nomenclature

STs	Steel fibers	nm	Nanometer
YRS	Yellow river sediment	#	Number
C-S-H	Calcium silicate hydrate	P	Pressure
C-A-S-H	Calcium aluminum silicate hydrate	XRD	X-ray Diffraction
ITZs	Interfacial Transition Zones	m	Meter
N	Newton	SiO_2_	Silicon Dioxide
PVAs	Polyvinyl Alcohol fibers	g	Gram
PEs	Polyethylene fibers	g cm^−3^	Grams per Cubic Centimeter
MPa	Megapascal	${SO}_{4}^{2-}$	Sulfate lon
OPC	Ordinary Portland cement	N	Newton
TGA	Thermogravimetric Analysis	SiO_2_	Silicon dioxide
SEM	Scanning electron microscope	SG	Specific gravity
mm	Millimeter	SS	Sodium Silicate
m^2^	Square meters	μW	Microwatt
m^2^ kg^−1^	Square meters per kilogram	NaOH	Sodium Hydroxide
min	Minute	%	Percentage
